# Imaging of Facial Muscles in Facioscapulohumeral Muscular Dystrophy: An Exploratory Study Comparing Magnetic Resonance Imaging and Ultrasound

**DOI:** 10.1002/mus.70360

**Published:** 2026-08-05

**Authors:** Federico Pistoia, Elena Faedo, Marta Macciò, Giovanni Marcenaro, Maria Elena Susi, Mehrnaz Hamedani, Veronica Caramellino, Sara Massucco, Andrea Francesco Maria Vollaro, Paola Mandich, Fabio Gotta, Riccardo Picasso, Federico Zaottini, Marina Grandis, Carlo Martinoli

**Affiliations:** ^1^ IRCCS Ospedale Policlinico San Martino Genoa Italy; ^2^ Department of Neurosciences, Rehabilitation, Ophthalmology, Genetic and Maternal and Infantile Sciences (DINOGMI) University of Genoa Genoa Italy; ^3^ Department of Health Sciences (DISSAL) University of Genoa Genoa Italy; ^4^ University of Genoa Genoa Italy

**Keywords:** facial imaging, facial weakness, facioscapulohumeral muscular dystrophy, MRI, ultrasound

## Abstract

**Aims:**

Facioscapulohumeral muscular dystrophy (FSHD) is a genetic progressive muscle disorder often presenting with facial weakness. However, imaging studies specifically evaluating facial muscle involvement and its relationship with clinical severity remain limited. This preliminary study explored magnetic resonance imaging (MRI) and ultrasound (US) assessment of facial muscle involvement and correlations with clinical severity.

**Methods:**

This single‐center prospective study enrolled 12 genetically confirmed FSHD1 patients (mean age 57.8 years). Clinical evaluation included the FSHD score, Comprehensive Clinical Evaluation Form (CCEF), and Facial Weakness Score (FWS). Muscle thickness of the orbicularis oris (OOr) and zygomaticus major (ZMj) was measured on both MRI and US, while MRI was additionally used for qualitative assessment of muscle bulk. Inter‐rater reliability was assessed for imaging measurements.

**Results:**

MRI and US showed comparable facial muscle thickness, with mean ZMj thickness of 2.34 ± 0.75 mm and 2.12 ± 0.55 mm, and OOr thickness of 2.28 ± 0.70 mm and 2.20 ± 0.67 mm, respectively. Inter‐rater reliability for MRI was high (ICC = 0.84), and reduced ZMj thickness showed moderate to strong correlation with greater disease severity (*ρ* up to 0.87, *p* < 0.001) and impaired facial gestures (*ρ* = 0.50–0.77, *p* < 0.05).

**Discussion:**

Despite the small sample size, MRI, and US assessment of facial muscles correlated with clinical severity and functional outcomes in FSHD, supporting their use as complementary tools. Longitudinal studies are required to establish imaging's role in monitoring changes over time.

**Abbreviations**
CCEF: comprehensive clinical evaluation form
CCS: clinical composite score
FOV: field of view
FSDH: facioscapulohumeral muscular dystrophy
FWS: facial weakness score
ICC: intraclass correlation coefficient
MRI: magnetic resonance imaging
NGEU: Next Generation European Union
OOr: orbicularis oris
SD: standard deviation
TSE: turbo spin‐echo
US: ultrasound
ZMj: zygomaticus major

## 1 Introduction

While limb magnetic resonance imaging (MRI) is used for diagnosis and monitoring facioscapulohumeral muscular dystrophy (FSHD), facial muscle involvement remains understudied despite its functional and communicative impact [1, 2, 3]. Objective strength evaluation of facial muscles is not feasible; assessment is usually indirect and based on the ability to perform facial expressions. In this context, imaging may provide more objective, muscle‐specific information. Although facial muscle echogenicity has been assessed with ultrasound (US) [4], the role of MRI remains undefined. The aim of our report was to explore FSHD facial muscle involvement using MRI, and to provide preliminary correlations with US and functional assessments.

## 2 Methods

This single‐center prospective study was approved by the local Ethics Committee (Registry number 41/2024 CER Liguria), and written informed consent was obtained from all study participants. Study participants with genetically confirmed FSHD and older than 18 years were enrolled at our institution between March 2024 and May 2025. Patients with a history of facial surgery, concomitant conditions affecting the facial muscles, or contraindications to MRI were excluded from the study.

### 2.1 Clinical Examination

Patients were classified using the Comprehensive Clinical Evaluation Form (CCEF) [5], a clinical tool that categorizes FSHD phenotypes based on the distribution and severity of muscle weakness. A detailed description of the CCEF classification is provided in Figure S1. Clinical evaluations were performed by a specialized neuromuscular team including a senior neurologist (M.G.), a neurology resident (E.F.), a physiotherapist (M.H.), and a final‐year medical student (V.C.). CCEF classification (Categories A–D; Figure S1) and the FSHD Clinical Score [5, 6], including assessment of horizontal smile and puckered lips (labial configuration resulting from the attempt to protrude lips as defined by Ricci et al. [7]), were assigned by a fixed rater pair (M.G. and E.F.). Facial involvement was further assessed using the Facial Weakness Score [3] (FWS) (2; 0 = normal, 21 = maximum severity; Table S1) by a second fixed rater pair (M.H. and V.C.). All evaluations were conducted using a consensus approach, blinded to imaging findings and to the results of other clinical assessments; disagreements were resolved by discussion based on predefined scoring criteria.

### 2.2 MRI Protocol

MRI of the facial muscles was performed using a 3 T Magnetom Prisma scanner equipped with a 64‐channel coil. Initial localizer scans were acquired with a wide field of view of 39.1 × 19.9 cm^2^ to ensure complete visualization of both sides of the face. The imaging protocol consisted of axial turbo spin‐echo T1‐weighted and T2‐weighted sequences with chemical shift selective fat saturation. Both sequences had a slice thickness of 3.0 mm and an interslice gap of 0.3 mm. Targeted FOVs of 170 (for T1) and 220 mm (for T2) were utilized to optimize spatial resolution. The in‐plane voxel size was 0.53 × 0.53 mm^2^ for T1‐weighted and 0.49 × 0.49 mm^2^ for T2‐weighted sequences. The total scan time was approximately 8 min (Table S2). One experienced musculoskeletal radiologist (F.P.) and two radiology residents with specific expertise in musculoskeletal imaging (M.M. and G.M.) independently assessed the ZMj, and OOr muscles. T2‐weighted images were reviewed for potential edema or abnormal signal. Muscle bulk was assessed using a composite visual scoring system specifically developed for this study which combined T1‐weighted signal characteristics with muscle thickness, adapting elements from existing grading systems and previously published thickness thresholds [8, 9, 10, 11]. The score was designed to integrate qualitative (T1‐weighted signal) and quantitative (muscle thickness measurements) parameters, based on the rationale that these two features may provide complementary information in the assessment of small mimetic muscles. The OOr and ZMj muscles were evaluated bilaterally for maximal thickness and fatty infiltration. A signal‐intensity score was assigned based on T1‐weighted appearance (0 = normal low signal; 1 = moderate hyperintense fatty infiltration; 2 = severe hyperintense fatty infiltration). Muscle thickness was incorporated as a binary modifier (0 = thickness≥reference threshold; 1 = thickness<reference threshold), resulting in a final composite score ranging from 0 to 3. Reference‐based thickness thresholds of 2.1 mm for ZMj and 1.7 mm for OOr were adopted from a previous US study to aid consistency in muscle classification [8]. As established MRI‐specific reference values for these muscles are currently unavailable, these thresholds were calibrated for the MRI scale by accounting for the mean inter‐modality offset observed in our sample, resulting in MRI‐adjusted thresholds of 2.04 for ZMj and 1.60 mm for OOr. Muscles were then classified into three classes: Class 1 (score 0–1, *normal*/*mild atrophy*), Class 2 (score 2, *moderate atrophy*), and Class 3 (score 3, *severe atrophy*). MRI‐derived muscle thickness values were compared with corresponding US measurements.

### 2.3 US Protocol

US examinations were performed with an Aplio i800 24–8 MHz linear array probe (Canon Medical System, Ōtawara, Japan). Study participants were examined in the supine position with the head in a neutral, straight orientation, consistent with MRI positioning and with the mouth closed. All examinations were performed by a single radiologist (F.P.), who acquired and archived standardized images during the same session; the examiner was blinded to MRI findings and clinical assessment results at the time of image acquisition. The examiner was seated to the patient's right side, and all acquisitions were performed bilaterally. The probe was placed perpendicular to the skin and transverse to the muscle fibers at standardized anatomical landmarks, with angle adjustments to avoid anisotropy and minimal pressure to prevent tissue compression. For the orbicularis oris, the probe was placed in a longitudinal orientation with its center positioned over the oral commissure. For the zygomaticus major, the muscle was visualized in its short axis by placing the probe in the transverse plane over the inferior portion of the zygomatic process. Detailed probe positions and the corresponding US appearances are illustrated in Figure S2.

### 2.4 Thickness Measurements

Maximal muscle thickness was measured electronically using the institutional PACS caliper tool (Carestream Vue; Carestream Health Inc. Rochester, NY, USA) by F.P., M.M., and G.M., all blinded to clinical data and to each other's evaluations. Thickness was defined as the maximum perpendicular distance between the inner and outer borders of the muscle, excluding subcutaneous fat. For US, each operator selected, among the previously stored images, the point of maximal muscle thickness for measurement. For MRI, each operator selected the axial T1‐weighted image showing the largest cross‐sectional area of the muscle for measurement. Measurements were performed independently with no consensus or adjudication. For descriptive and correlation analyses, muscle thickness values were pooled by averaging the three observers' measurements for each muscle and modality.

### 2.5 Statistical Analysis

The statistical analysis included an initial exclusion of variables with near‐zero variance. Subsequently, pairwise associations between variables were evaluated using Spearman's rank correlation coefficient (*ρ*). Statistical significance was assessed using the exact permutation test for the *p*‐value, with a threshold of *p* < 0.05 considered statistically significant. 95% confidence intervals for Spearman correlation coefficients were computed using bootstrap resampling. All imaging measurements, including muscle thickness and signal‐intensity scoring, were independently performed by three observers blinded to clinical data. Following the inter‐observer reliability assessment, all reported measurements represent the mean of the three independent assessments. Inter‐observer agreement was assessed using the intraclass correlation coefficient (ICC) for continuous variables, employing a two‐way random‐effects model for absolute agreement. For ordinal categorical variables, agreement was quantified using the weighted Cohen's kappa (with squared weights). Both ICC and weighted kappa were reported with their corresponding 95% confidence intervals. All statistical analyses were performed using RStudio (version 4.4.2; R Foundation for Statistical Computing, Vienna, Austria).

## 3 Results

### 3.1 Clinical Characteristics

Twelve genetically confirmed FSHD1 patients were included in the study (8 males; 66.7%), with a mean age of 57.8 years (SD: 14.3, range: 25–75). Six participants were classified as Category A, including A1 (*n* = 2; one male) and A2 (*n* = 2; two males), while Category B included one male in B1 and one female in B2. Finally, six participants fell into Category D1 (four males) (Table 1; Tables S3–S5; Figure S3).

**TABLE 1:** Demographic and clinical characteristics of the study cohort (*n* = 12).

Variable	Mean	SD	Median	Min	Max
Age at evaluation (years)	57.8	14.3	59	25	76
Age at onset (years)	33.8	23.3	16	12	68
Duration of disease (years)	23.8	16.9	20	3	49
FSHD clinical score	7.58	2.47	8.00	4.00	12.00
FWS	6.80	3.98	8.00	0.00	12.00

*Note:* Values are reported as mean, standard deviation (SD), median, and range (minimum–maximum).

### 3.2 Imaging Findings (MRI and US)

**FIGURE 1:**
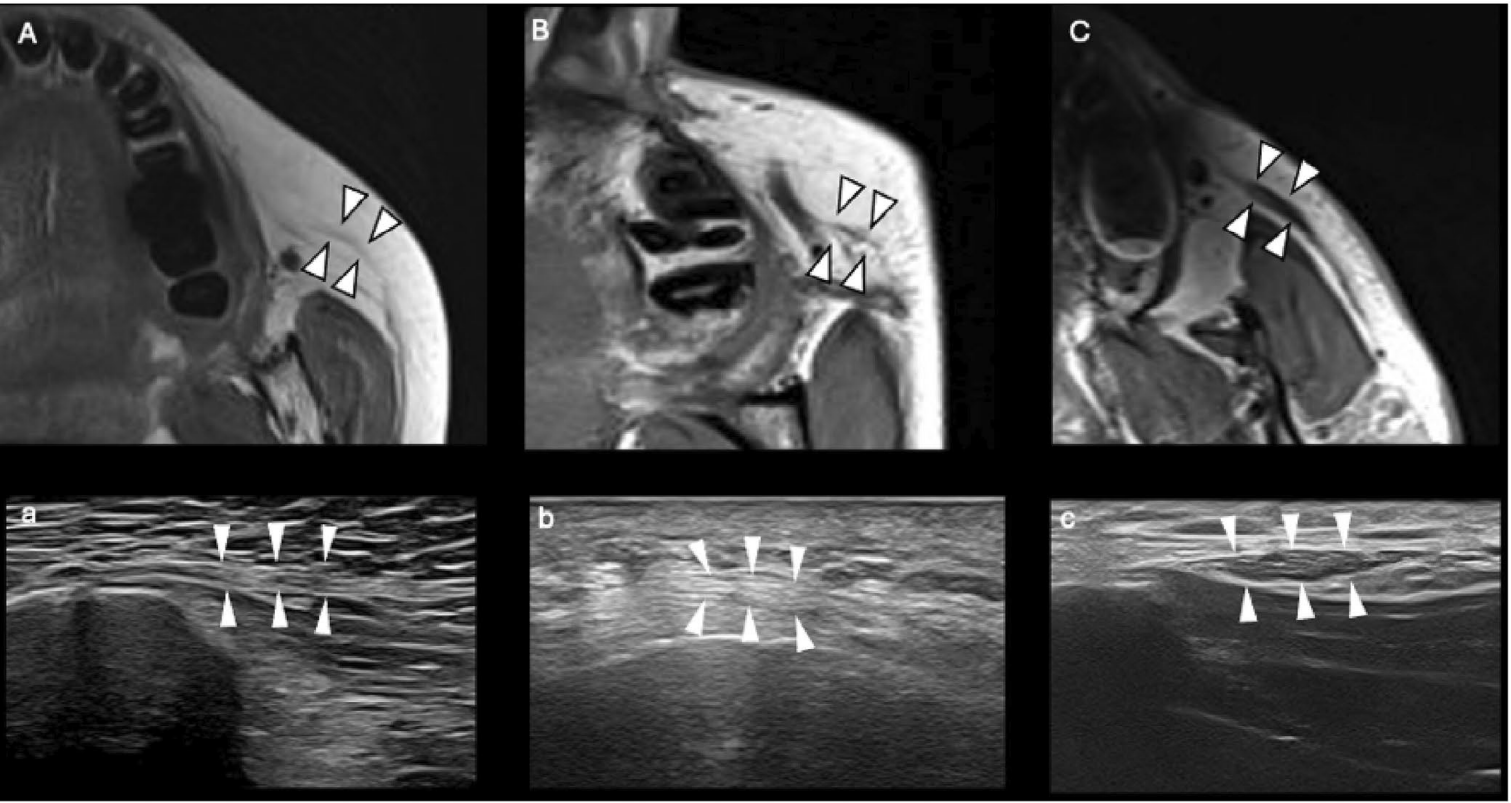
Axial TSE T1‐weighted MRI and transverse high‐frequency US (24–8 MHz) images of the ZMj muscle (black‐lined white arrowhead in MRI imaging and white arrowheads in US imaging) in three study participants. (A and a) In a 26‐year‐old patient, the ZMj shows advanced atrophy with near‐complete fatty replacement (bulk score 3). The muscle appears markedly thinned and nearly isointense to subcutaneous fat on T1‐weighted MRI (A, black‐lined white arrowheads), and thinned and hyperechoic on US (a, white arrowheads). (B and b) In a 59‐year‐old patient, moderate atrophic changes are present, with partial fatty infiltration (bulk score 2). The ZMj muscle remains partially hypointense on T1‐weighted MRI (B, black‐lined white arrowheads) and appears thinned and hyperechoic on US (b, white arrowheads). (C and c) In a 61‐year‐old patient, the ZMj shows preserved morphology and signal characteristics (bulk score 1), appearing thick and hypointense relative to subcutaneous tissue on T1‐weighted MRI (C, black‐lined white arrowheads), and hypoechoic on US (c, white arrowheads). TSE, turbo spin‐echo; MRI, magnetic resonance imaging; US, ultrasound; ZMj, zygomaticus major.

**FIGURE 2:**
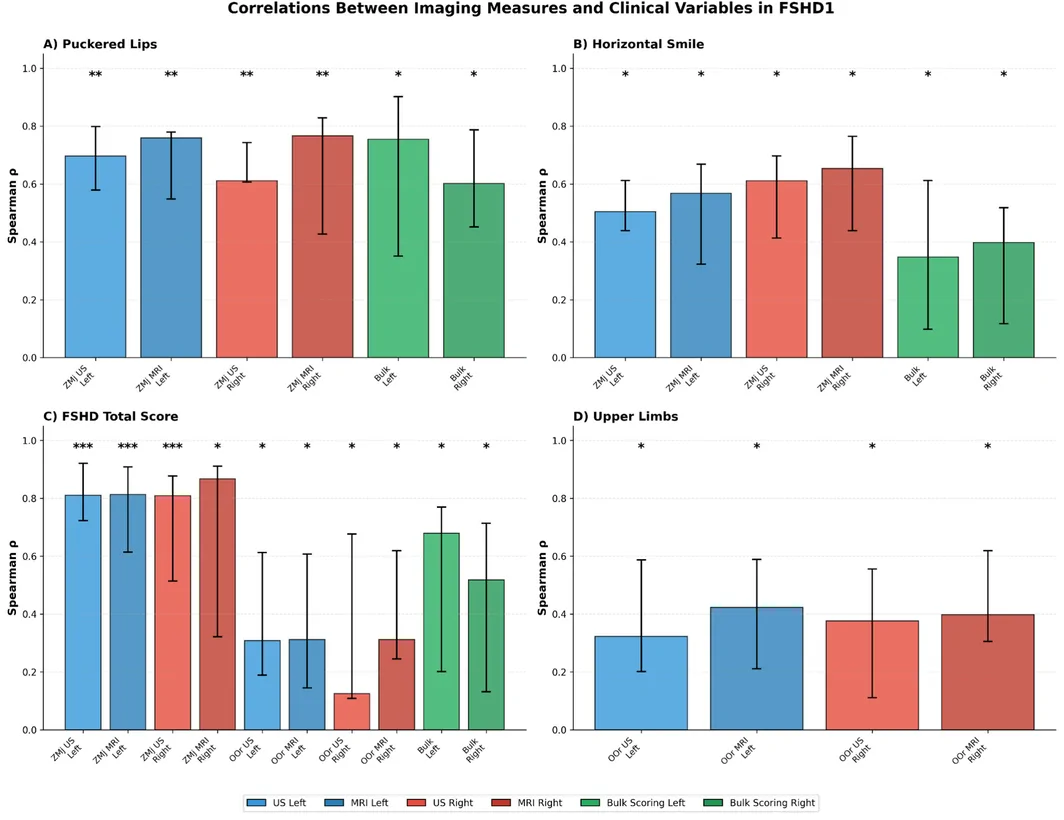
Correlations between facial muscle thickness and clinical variables in FSHD1 patients. Bar graphs showing Spearman correlation coefficients (*ρ*) between imaging measures and clinical variables in FSHD1 patients (*n* = 12). Panels display correlations for: (A) Puckered Lips, (B) Horizontal Smile, (C) FSHD total score, and (D) Upper Limbs. For each panel, bars represent the six imaging measures: Zygomaticus Major (ZMj) US Left, ZMj MRI Left, ZMj US Right, ZMj MRI Right, Orbicularis Oris (OOr) US Left, OOr MRI Left, OOr US Right, OOr MRI Right, and Bulk Score Left and Right. Error bars represent 95% bootstrap confidence intervals. Asterisks denote statistical significance: *p < 0.05, **p < 0.01, ***p < 0.001. ZMj, zygomaticus major; MRI, magnetic resonance imaging; US, ultrasound.

Mean ZMj thickness was 2.34 ± 0.75 on MRI and 2.12 ± 0.55 mm on US; mean OOr thickness was 2.28 ± 0.70 on MRI and 2.20 ± 0.67 mm on US. Reduced ZMj thickness was significantly associated with impaired facial gestures, including puckered lips (*ρ* up to 0.76) and horizontal smile (*ρ* up to 0.65). OOr thickness showed weak‐to‐moderate correlations with upper limb impairment (*ρ* = 0.32–0.42). Figure 1 illustrates representative imaging findings of the ZMj muscle in three study participants. ZMj MRI scoring demonstrated strong correlations with puckered lips, moderate correlations with total FSHD score, and weak‐to‐moderate correlations with horizontal smile (Figure 2 and Table S6). On T2‐weighted sequences, no alterations were observed.

### 3.3 Correlations Between Modalities

Although no statistically significant differences were observed between MRI and US measurements, a small and consistent mean inter‐modality offset was observed. Accordingly, US‐derived reference thresholds were translated to MRI‐specific values (2.04 for ZMj and 1.60 mm for OOr) for MRI‐based scoring.

## 4 Discussion

Despite the limited sample size, good inter‐observer agreement was observed, and no significant differences emerged between MRI‐ and US‐derived thickness measurements, suggesting that US may represent a viable and more accessible alternative for this parameter.

The composite MRI bulk scoring system introduced in this study integrates qualitative and quantitative parameters, namely the assessment of fatty infiltration on T1‐weighted images and muscle thickness. This choice was motivated by evidence from US indicating that muscle thickness and qualitative markers of degeneration provide complementary information; specifically, Vincenten et al. demonstrated that increased echogenicity and reduced thickness do not always overlap in FSHD, suggesting a potential dissociation between these biomarkers [12]. We hypothesized that a similar dissociation could be captured with MRI, where T1‐weighted fatty infiltration and muscle thickness might independently reflect different aspects of facial muscle involvement. Integrating both dimensions into a composite MRI score could therefore offer a more comprehensive assessment of the total disease burden, particularly in small mimetic muscles where isolated parameters may be less informative. Although this exploratory approach showed promising correlations with clinical severity in our cohort, the added value of the composite score over single‐parameter assessment requires confirmation in larger studies.

The high US reproducibility should be interpreted with caution, as image acquisition was performed by a single operator and measurements were obtained by three experienced raters in interpreting this anatomical region, which may have reduced variability and inflated inter‐observer agreement. Thickness thresholds used were not intended as validated cut‐off values of normality. They served as reference‐based operational thresholds to support visual classification, and were expressed on the MRI scale to ensure modality‐consistent interpretation. No statistically significant differences were observed between MRI and US thickness measurements. This likely reflects the influence of partial volume effects inherent to the 3.0 mm slice thickness used in our MRI protocol. When a muscle thinner than 3.0 mm occupies only a portion of the 3.0‐mm voxel in the through‐plane direction, the signal from adjacent subcutaneous fat or perimuscular connective tissue is averaged within the same voxel. This effect can blur the muscle‐fat interface, potentially leading to a slight overestimation of apparent muscle thickness on MRI. Conversely, US provides superior sub‐millimetric in‐plane resolution, allowing for a more direct and precise resolution of these interfaces. These differences have two practical implications: first, MRI‐derived thickness values might slightly overestimate true muscle bulk due to signal averaging at the edges; second, the MRI bulk scoring system could potentially underclassify atrophy severity (i.e., appearing less severe) in cases where partial volume averaging makes a thinned muscle appear thicker than it is. These points reinforce the exploratory nature of the current scoring system and the need for future validation using thinner slices or higher‐field acquisitions. Facial muscles are small, making the difference in spatial resolution between US and MRI particularly relevant: US, with its higher resolution, may detect subtle early changes, whereas MRI becomes more informative in later stages, as it can reliably distinguish fatty infiltration from muscle edema [5, 6, 7, 8, 9]. Our findings extend previous work showing that US and MRI can reliably demonstrate facial muscles and their thickness [4, 13, 14, 15, 16, 17]. More recently, Vincenten et al. used US to characterize orofacial involvement in a large FSHD cohort, demonstrating frequent echogenicity changes and weakness in several mimetic and masticatory muscles and supporting the feasibility and reliability of quantitative facial muscle US in this disease [4]. Our results show that reduced ZMj bulk scoring and thickness correlates with facial weakness and FSHD total score (Table S6), complementing the findings reported by Vincenten et al. [4].

In contrast to Vincenten et al. [4], we did not assess echo intensity (EI), as EI is highly sensitive to acquisition and analysis settings and would have added methodological complexity in this small, multi‐rater MRI‐comparison study [18]. Given the small size and complex anatomy of facial muscles, automated volumetric MRI analysis and DTI were considered technically challenging due to partial volume effects [19, 20, 21, 22]. Accordingly, this study focused on practical, reproducible measures (signal‐intensity scoring and thickness), while more advanced techniques are deferred to larger future studies.

Given its preliminary nature, this study presents some expected limitations, including a small sample size, resulting in skewed score distributions and lack of healthy controls. Furthermore, the high proportion of D1 phenotypes in our cohort is likely an incidental finding driven by the small sample size, which may limit the generalizability of our findings to the broader FSHD phenotypic spectrum. Additionally, the reliance on visual scoring systems may introduce some subjectivity. Validation in larger cohorts, including healthy controls, is needed to confirm these findings and improve phenotypic stratification. While longitudinal data are required to assess disease monitoring, these preliminary results suggest that MRI and US provide complementary objective data for assessing facial involvement in FSHD. Moreover, the reported Spearman correlation coefficients may be subject to upward bias due to small‐sample instability and should therefore be interpreted with caution.

## Author Contributions

**Federico Pistoia:** conceptualization, methodology, writing – original draft, writing – review and editing. **Fabio Gotta:** conceptualization, methodology, writing – original draft, writing – review and editing. **Paola Mandich:** conceptualization, methodology, writing – original draft, writing – review and editing. **Federico Zaottini:** conceptualization, methodology, writing – review and editing, writing – original draft. **Marta Macciò:** conceptualization, methodology. **Giovanni Marcenaro:** conceptualization, methodology, writing – original draft, writing – review and editing. **Andrea Francesco Maria Vollaro:** conceptualization, methodology, writing – original draft, writing – review and editing. **Marina Grandis:** conceptualization, methodology, writing – original draft, writing – review and editing. **Maria Elena Susi:** conceptualization, methodology, writing – original draft, writing – review and editing. **Sara Massucco:** conceptualization, methodology, writing – original draft, writing – review and editing. **Elena Faedo:** conceptualization, methodology, writing – original draft, writing – review and editing. **Carlo Martinoli:** conceptualization, methodology, writing – original draft, writing – review and editing. **Riccardo Picasso:** conceptualization, methodology, writing – original draft, writing – review and editing. **Veronica Caramellino:** conceptualization, methodology, writing – original draft, writing – review and editing. **Mehrnaz Hamedani:** conceptualization, methodology, writing – original draft, writing – review and editing.

## Funding

This work was supported by the Ministry of University and Research (MUR) (DN. 1553 11.10.2022).

## Disclosure

We confirm that we have read the Journal's position on issues involved in ethical publication and affirm that this report is consistent with those guidelines.

## Conflicts of Interest

The authors declare no conflicts of interest.

## Supporting information


**Figure S1:** Decision‐making algorithm for clinical classification of suspected FSHD cases according to the Comprehensive Clinical Evaluation Form (CCEF).
**Figure S2:** (A) Ultrasound (US) appearance of the orbicularis oris muscle (white arrowheads) and (a) corresponding probe position in the longitudinal orientation, with the center of the probe placed over the oral commissure. (B) US image of the zygomaticus major muscle (white arrowheads) and (b) corresponding probe position in the transverse axis over the inferior portion of the zygomatic process.
**Figure S3:** Distribution of Facial Weakness Score (FWS) item impairment and characteristic facial signs in the FSHD1 cohort (*n* = 12).
**Table S1:** Facial Weakness Score (FWS) instructions.
**Table S2:** Magnetic Resonance Imaging acquisition parameters.
**Table S3:** Summary of the main demographic, clinical, and radiological continuous data of the study.
**Table S4:** Summary of the main demographic, clinical, and radiological categorial data of the study.
**Table S5:** Comparison of bulk values across FSHD categories.
**Table S6:** Correlations between imaging‐derived measurements of facial muscles (US and MRI) and clinical functional scores.

## Data Availability

The data that support the findings of this study are available from the corresponding author upon reasonable request.
