# Supplementary material for: Imaging of Facial Muscles in Facioscapulohumeral Muscular Dystrophy: An Exploratory Study Comparing Magnetic Resonance Imaging and Ultrasound
Source: Muscle Nerve. 2026 Aug 5;74(4):1256–62. doi: 10.1002/mus.70360 (PMC13549152; doi:10.1002/mus.70360)
Supplement: Supplementary file 1 [file MUS-74-1256-s001.docx]

**Supplemental Figure 1**: Decision-making algorithm for clinical classification of suspected FSHD cases according to the Comprehensive Clinical Evaluation Form (CCEF). The flowchart integrates scapular and facial muscle scores with the evaluation of typical features, guiding assignment to phenotypic categories A (typical FSHD with varying facial involvement), B (incomplete phenotypes: isolated scapular or facial weakness), C (non-penetrant carriers: minor signs or normal examination), and D (atypical myopathic presentations or characteristics).

**Supplemental Figure 2.** (A) Ultrasound (US) appearance of the orbicularis oris muscle (white arrowheads) and (a) corresponding probe position in the longitudinal orientation, with the center of the probe placed over the oral commissure. (B) US image of the zygomaticus major muscle (white arrowheads) and (b) corresponding probe position in the transverse axis over the inferior portion of the zygomatic process.

**Supplemental Figure 3:** Distribution of Facial Weakness Score (FWS) item impairment and characteristic facial signs in the FSHD1 cohort (n=12). Each bar represents the number of patients showing normal function (FWS 0), moderate weakness (FWS 1–2), or severe weakness (FWS 3) for each tested movement. “Horizontal smile” and “puckered lips” were scored separately as distinctive qualitative features of advanced facial involvement.

| **Facial Weakness Score (FWS)** | | | | |
| --- | --- | --- | --- | --- |
| **Region** | **Grade 0** | **Grade 1** | **Grade 2** | **Grade 3** |
| Mouth and forehead | Complete movement possible | Near complete movement possible | Limited movement possible | Impossible to initiate |
| Eye closure | Complete eye closure | Small rim of eyelashes visible | Eyelashes mostly visible | Incomplete eye closure |

**Supplemental Table 1**: Facial Weakness Score (FWS) instructions. For each region, Grade 0 indicates normal movement, while Grade 3 denotes a complete inability to perform the indicated action. Intermediate grades reflect varying degrees of movement limitation or incomplete closure, as specified.

| **Parameters** | **Axial TSE T1** | **Axial TSE T2 Fat Sat** |
| --- | --- | --- |
| **Slice thickness (mm)** | 3 | 3 |
| **TR (ms)** | 649 | 6860 |
| **TE (ms)** | 9.6 | 96 |
| **NEX (Number of Excitations)** | 2 | 2 |
| **Matrix size** | 320 | 448 |
| **FOV (cm^2^)** | 170 | 220 |
| **In-plane voxel size (mm^2^)** | 0.53x0.53 | 0.49x0.49 |

**Supplemental Table 2.** Magnetic Resonance Imaging acquisition parameters.

TSE: Turbo Spin Echo; TR: Repetition Time; TE: Echo Time; nex: Number of Excitations; FOV: Field of View; matrix: Acquisition Matrix; pixel: In-plane spatial resolution. The ~~%~~FOV represents the rectangular field of view percentage relative to the main FOV.

| **Variable** | **Mean** | **SD** | **Median** | **IQR** |
| --- | --- | --- | --- | --- |
| **Demographics** |  |  |  |  |
| Age at onset (yrs) | 33.75 | 23.28 | 25.50 | 12.75–54.25 |
| **FSHD clinical score** |  |  |  |  |
| Total score (0–15) | 7.58 | 2.47 | 8.00 | 5.8–9.0 |
| Facial subscore (0–2) | 1.00 | 0.43 | 1.0 | 1.0–1.0 |
| Scapular girdle subscore (0–3) | 1.67 | 1.07 | 2.0 | 1.5–2.0 |
| Upper limbs subscore (0–2) | 1.00 | 0.60 | 1.0 | 1.0–1.0 |
| Abdominal muscle subscore (0–1) | 0.67 | 0.49 | 1.0 | 0.0–1.0 |
| Pelvic girdle subscore (0–5) | 1.83 | 1.03 | 1.5 | 1.0–2.2 |
| Legs subscore (0–2) | 1.42 | 0.51 | 1.0 | 1.0–2.0 |
| CSS score (0–5) | 3.12 | 0.68 | 3.00 | 2.50–3.50 |
| **Facial Weakness Score (FWS)** |  |  |  |  |
| FWS total score (0–12) | 6.83 | 3.97 | 8.0 | 3.5–10.0 |
| Atypical features (0–3) | 0.92 | 1.08 | 0.5 | 0.0–2.0 |
| Closing eye gently (0–3) | 0.58 | 0.90 | 0.0 | 0.0–1.0 |
| Closing eyes firmly (0–2) | 0.67 | 0.78 | 0.5 | 0.0–1.0 |
| Raising eyebrow (0–2) | 0.58 | 0.67 | 0.5 | 0.0–1.0 |
| Frowning (0–2) | 1.17 | 0.72 | 1.0 | 1.0–2.0 |
| Pursing lips (0–2) | 1.33 | 0.89 | 2.0 | 0.8–2.0 |
| Showing teeth (0–2) | 1.25 | 0.87 | 1.5 | 0.8–2.0 |
| Puffing cheeks (0–2) | 1.17 | 0.83 | 1.0 | 0.8–2.0 |
| **Imaging measurements (n = 24 sides)** |  |  |  |  |
| OOr thickness — MRI (mm) | 2.28 | 0.70 | 2.20 | 1.91–2.89 |
| ZMj thickness — MRI (mm) | 2.34 | 0.75 | 2.20 | 1.96–3.14 |
| OOr thickness — US (mm) | 2.20 | 0.67 | 2.11 | 1.85–2.54 |
| ZMj thickness — US (mm) | 2.12 | 0.55 | 2.08 | 1.92–2.37 |

**Supplemental Table 3**. Summary of the main demographic, clinical, and radiological continuous data of the study.

| **Variable** | **Grade** | **n** | **%** |
| --- | --- | --- | --- |
| **Facial characteristics** |  |  |  |
| Puckered lips | No | 7 | 58.3 |
|  | Yes | 5 | 41.7 |
| Horizontal smile | Yes | 7 | 58.3 |
|  | No | 5 | 41.7 |
| **FSHD clinical score - grade distribution** |  |  |  |
| Facial subscore (0–2) | 0 | 1 | 8.3 |
|  | 1 | 10 | 83.3 |
|  | 2 | 1 | 8.3 |
| Scapular girdle subscore (0–3) | 0 | 3 | 25.0 |
|  | 2 | 7 | 58.3 |
|  | 3 | 2 | 16.7 |
| Upper limbs subscore (0–2) | 0 | 2 | 16.7 |
|  | 1 | 8 | 66.7 |
|  | 2 | 2 | 16.7 |
| Abdominal muscle subscore (0–1) | 0 | 4 | 33.3 |
|  | 1 | 8 | 66.7 |
| Pelvic girdle subscore (0–5) | 1 | 6 | 50.0 |
|  | 2 | 3 | 25.0 |
|  | 3 | 2 | 16.7 |
|  | 4 | 1 | 8.3 |
| Legs subscore (0–2) | 1 | 7 | 58.3 |
|  | 2 | 5 | 41.7 |
| **FWS items - grade distribution** |  |  |  |
| Atypical features (0–3) | 0 | 6 | 50.0 |
|  | 1 | 2 | 16.7 |
|  | 2 | 3 | 25.0 |
|  | 3 | 1 | 8.3 |
| Closing eye gently (0–3) | 0 | 7 | 58.3 |
|  | 1 | 4 | 33.3 |
|  | 3 | 1 | 8.3 |
| Closing eyes firmly (0–2) | 0 | 6 | 50.0 |
|  | 1 | 4 | 33.3 |
|  | 2 | 2 | 16.7 |
| Raising eyebrow (0–2) | 0 | 6 | 50.0 |
|  | 1 | 5 | 41.7 |
|  | 2 | 1 | 8.3 |
| Frowning (0–2) | 0 | 2 | 16.7 |
|  | 1 | 6 | 50.0 |
|  | 2 | 4 | 33.3 |
| Pursing lips (0–2) | 0 | 3 | 25.0 |
|  | 1 | 2 | 16.7 |
|  | 2 | 7 | 58.3 |
| Showing teeth (0–2) | 0 | 3 | 25.0 |
|  | 1 | 3 | 25.0 |
|  | 2 | 6 | 50.0 |
| Puffing cheeks (0–2) | 0 | 3 | 25.0 |
|  | 1 | 4 | 33.3 |
|  | 2 | 5 | 41.7 |
| **MRI ordinal scores (n = 24 sides)** |  |  |  |
| ZMj signal intensity (1–2) | 1 | 22 | 91.7 |
|  | 2 | 2 | 8.3 |
| ZMj bulk score (1–3) | 1 | 15 | 62.5 |
|  | 2 | 5 | 20.8 |
|  | 3 | 4 | 16.7 |

**Supplemental Table 4**. Summary of the main demographic, clinical, and radiological categorial data of the study.

| FSHD Category | N (patients) | ZM Bulk Score (MRI bulk) |
| --- | --- | --- |
| A1 | 2 | 1.83 ± 1.18 |
| A2 | 2 | 1.33 ± 0.47 |
| B1 | 1 | 1.33 |
| B2 | 1 | 1.17 |
| D1 | 6 | 1.33 ± 0.82 |

**Supplememental Table 5.** Comparison of bulk values across FSHD categories.

| **Imaging Measure** | **Clinical Score** | **\|ρ\|** | **95% CI** | **p-value** |
| --- | --- | --- | --- | --- |
| ZMj thickness US left | Puckered lips | 0.697 | [0.579; 0.799] | <0.01 ** |
| ZMj thickness MRI left | Puckered lips | 0.759 | [0.548; 0.779] | <0.01 ** |
| ZMj thickness US right | Puckered lips | 0.611 | [0.507; 0.743] | <0.01 ** |
| ZMj thickness MRI right | Puckered lips | 0.766 | [0.427; 0.829] | <0.01 ** |
| ZMj thickness US left | Horizontal smile | 0.504 | [0.439; 0.612] | <0.05 * |
| ZMj thickness MRI left | Horizontal smile | 0.568 | [0.323; 0.668] | <0.05 * |
| ZMj thickness US right | Horizontal smile | 0.611 | [0.413; 0.697] | <0.05 * |
| ZMj thickness MRI right | Horizontal smile | 0.654 | [0.439; 0.765] | <0.05 * |
| ZMj thickness US left | FSHD total score | 0.811 | [0.723; 0.921] | <0.001 *** |
| ZMj thickness MRI left | FSHD total score | 0.813 | [0.614; 0.909] | <0.001 *** |
| ZMj thickness US right | FSHD total score | 0.809 | [0.514; 0.877] | <0.001 *** |
| ZMj thickness MRI right | FSHD total score | 0.867 | [0.321; 0.911] | <0.05 * |
| OOr thickness US left | FSHD total score | 0.308 | [0.189; 0.613] | <0.05 * |
| OOr thickness MRI left | FSHD total score | 0.312 | [0.145; 0.607] | <0.05 * |
| OOr thickness US right | FSHD total score | 0.225 | [0.109; 0.677] | <0.05 * |
| OOr thickness MRI right | FSHD total score | 0.312 | [0.245; 0.619] | <0.05 * |
| OOr thickness US left | Upper limbs | 0.323 | [0.201; 0.587] | <0.05 * |
| OOr thickness MRI left | Upper limbs | 0.423 | [0.211; 0.589] | <0.05 * |
| OOr thickness US right | Upper limbs | 0.376 | [0.111; 0.556] | <0.05 * |
| OOr thickness MRI right | Upper limbs | 0.398 | [0.305; 0.619] | <0.05 * |
| ZMj bulk scoring MRI left | Puckered lips | 0.755 | [0.351; 0.902] | <0.05 * |
| ZMj bulk scoring MRI right | Puckered lips | 0.602 | [0.452; 0.787] | <0.05 * |
| ZMj bulk scoring MRI left | Horizontal smile | 0.348 | [0.098; 0.612] | <0.05 * |
| ZMj bulk scoring MRI right | Horizontal smile | 0.398 | [0.117; 0.518] | <0.05 * |
| ZMj bulk scoring MRI left | FSHD total score | 0.679 | [0.201; 0.688] | <0.05 * |
| ZMj bulk scoring MRI right | FSHD total score | 0.518 | [0.132; 0.714] | <0.05 * |

* p < 0.05 · ** p < 0.01 · *** p < 0.001

**Supplemental Table 6.** Correlations between imaging-derived measurements of facial muscles (ultrasound and MRI) and clinical functional scores. Spearman’s correlation coefficients (|ρ|), 95% confidence intervals (CI), and p-values are reported. ZMj = zygomaticus major; OOr = orbicularis oris; US = ultrasound; MRI = magnetic resonance imaging; FSHD = facioscapulohumeral muscular dystrophy. Statistical significance is indicated as *p < 0.05 and **p < 0.01.*
